# Molecular Differentiation and Detection of AMR Genes from Nosocomial *Staphylococcus* spp.

**DOI:** 10.3390/pathogens14050403

**Published:** 2025-04-23

**Authors:** Isabelle Carneiro, Wellington Luiz Pontes da Silva, Debora Ribeiro de Souza Santos, Ivano de Filippis

**Affiliations:** National Institute for Quality Control in Health, Oswaldo Cruz Foundation—INCQS/FIOCRUZ, Rio de Janeiro 21040-900, Brazil; isabellecgs10@gmail.com (I.C.);

**Keywords:** *Staphylococcus aureus*, *Staphylococcus haemolyticus*, MRS, MDR, qPCR-HRM

## Abstract

*Staphylococcus* spp. is a major nosocomial pathogen, particularly affecting immunocompromised patients and infants. It is associated with bacteremia, endocarditis, and co-infections. Methicillin-resistant *Staphylococci* (MRS) carry the *mecA* gene, encoding PBP2a, which confers resistance to beta-lactam antibiotics. The aim of this study is to investigate resistance profiles and develop a molecular method to identify nosocomial *Staphylococcus* spp. strains. A total of 64 strains from public hospitals in Rio de Janeiro were analyzed using phenotypic and molecular methods, with 17 classified as MDR. Different melting temperatures (Tm) were obtained through qPCR-HRM analysis, to identify *S. aureus-* (70.4 °C), *S. haemolyticus-* (79 °C), *S. epidermidis-* (74.1 °C) and *mecA* (70.5 °C)-positive strains (MRS). The *mecA* gene was detected in 51 strains, with 22 showing SCCmec type IV. The spread of MRSA and MDR *Staphylococci*, particularly MDR *S. haemolyticus*, is a growing concern. In our study, among 64 Staphylococci strains, only 11 were susceptible to methicillin, showing the continuous emergence of resistant strains. qPCR-HRM is a cost-effective, sensitive and fast method for rapid *Staphylococcus* spp. identification, aiding in nosocomial infection control.

## 1. Introduction

According to the List of Prokaryotic names with Standing in Nomenclature (LPSN), the *Staphylococcus* genus has approximately 89 species (accessed 23 February 2025) [1].

*Staphylococcus aureus* is listed in the ESKAPE group (*Enterococcus faecium*, *Staphylococcus aureus*, *Klebsiella pneumoniae*, *Acinetobacter baumannii*, *Pseudomonas aeruginosa*, *Escherichia coli*) and is the main pathogen involved with nosocomial and community-associated infections, particularly methicillin-resistant variants (MRSA) [2,3]. Although this genus has been known as an opportunistic microorganism, for many years, Coagulase-Negative *Staphylococcus* (CoNS) have long been recognized as opportunistic pathogens in immunocompromised patients and device-associated infections present in the human microbiota [4]. However, their pathogenicity has been proven due to their constant association with infections, such as bacteremia and high resistance levels to antimicrobials [5]. *Staphylococcus haemolyticus* is the second most frequently isolated in nosocomial infections, with Staphylococcus epidermidis being most prevalent among coagulase-negative *Staphylococcus* (CoNS). The *S. haemolyticus* bacteria may cause severe infections on the skin and in the urinary tract, as well as in prosthetic joints [6].

Frequently, the resistance of *S. haemolyticus* to antimicrobials is associated with methicillin- and multidrug-resistant strains. In addition, the bacteria play an important role as a reservoir of antimicrobial resistant genes due to its genomic plasticity; therefore, it is responsible to carry more than one plasmid in a cell, allowing their widespread dissemination within the genus [7].

The methicillin-resistant staphylococci (MRS) carry the *mecA* gene, which encodes the synthesis of PBP2a, a protein that exhibits a low affinity for beta-lactams. Staphylococcal Cassette Chromosome *mec* (SCCmec) is a large, site-specific and horizontally transferable genomic island composed of several key elements. The *mecA* gene could be carried on SCCmec, allowing its transfer among staphylococcal species [8]. During the COVID-19 pandemic, due to the indiscriminate use of antimicrobial agents, studies have reported innumerous infections by MRSA and MRCoNS with a prevalence of *S. haemolyticus* emerging, associated with neonatal bloodstream infections due to transmission during labor [9].

Due to similar biochemical and coagulase test reactions, phenotypic methods may lead to misidentification between coagulase-positive and coagulase-negative species. Coagulase activity decreases in vancomycin-resistant *Staphylococcus aureus* (VRSA), possibly because of exhibiting cell wall thickening or low expression of plasma coagulation enzyme gene, and the presence of the *coa* gene in CoNS may be acquired by mobile genetic elements from coagulase-positive *Staphylococcus* [10,11].

The aim of this study is to investigate the resistance profile of *Staphylococcus* spp. strains and develop a molecular protocol for cost-effective and accurate discrimination among *S. aureus*, *S. haemolyticus*, *S. epidermidis* and MRS strains, isolated from nosocomial samples in Rio de Janeiro city, Brazil.

## 2. Materials and Methods

### 2.1. Clinical and Control Strains

A total of 64 strains were analyzed in this study. The isolates were obtained from patients with nosocomial infections from public hospitals in Rio de Janeiro. The strains, previously identified by VITEK II (BIOMERIEUX, Lyon, France) and confirmed at our laboratory, were recovered from blood cultures *n* = 30 (46.8%), nasal swab *n* = 22 (34.3%), urine *n* = 4 (6.25%), ulcer swab *n* = 1 (1.5%), abscess *n* = 2 (3.1%), subcutaneous liquid *n* = 1 (1.5%), mammary secretion *n* = 1 (1.5%), wound secretion *n* = 1 (1.5%), and two strains’ origins were not determined (3.1%).

An additional 10 strains were obtained from the *Pathogenic Bacteria Collection* (CBP) of the *Instituto Nacional de Controle de Qualidade em Saúde*—INCQS/FIOCRUZ and were used as reference in this study. Control strains used were *S. saprophyticus* CBP 00233 (ATCC15305), *S. warneri* CBP 00243 (ATCC 10209), *S. hominis* (Type strain) CBP 00359 (ATCC 27844), *S. aureus* CBP 00577 (ATCC 43300), *S. aureus* CBP 00612 (ATCC 700698), *S. aureus* CBP 00613 (ATCC 700699), *S. aureus* (MRSA) CBP 00306 (ATCC 33591), *S. aureus* CBP 00307 (ATCC 33592), *S. aureus* (Type Strain) CBP 00358 (ATCC 12600) and *S. epidermidis* CBP 00016 (ATCC 12228). We were not able to obtain a strain of *S. haemolyticus* to be used as reference; thus, strain 98 obtained from a blood culture, identified as *S. haemolyticus* by VITEK II (BIOMERIEUX, Lyon, France) with 99% similarity, was used as a positive control strain.

### 2.2. Phenotypic Identification

Clinical samples were seeded on nutrient and mannitol salt agar plates and incubated at 37 °C for 24 h and subjected to Gram staining and subsequent coagulase test, using strain *S. aureus* CBP 00358 as positive control. Isolate identification was performed using VITEK II following the manufacturer’s instructions.

### 2.3. Antimicrobial Susceptibility Tests

The Kirby–Bauer disk diffusion method was implemented for the analysis of antimicrobial susceptibility. Strains identified as *Staphylococcus* spp. were seeded on Mueller–Hinton agar and evaluated with the following antimicrobial disks: penicillin (10 µg), oxacillin (1 µg) and vancomycin (5 µg). Plates were incubated at 37 °C for 24 h and the results were interpreted according to the Clinical and Laboratory Standards Institute (CLSI) guidelines. Minimum inhibitory concentrations (MICs) were determined by VITEK II, revealing several multidrug-resistant (MDR) strains.

### 2.4. Identification by qPCR with High-Resolution Melting (qPCR-HRM)

For all molecular procedures, genomic DNA extraction was carried out with the *PureLink Genomic DNA Mini Kit* (Invitrogen) according to the manufacturer’s instructions.

The identification of *Staphylococcus* spp. species by qPCR-HRM was performed in a *QuantStudio7 Flex Real-Time PCR System*, (Applied Biosystems, Foster City, CA, USA), using the primers listed in Table 1. Reference strains were *S. aureus* INCQS 00358 (ATCC 12600), *S. haemolyticus* CBP 98, *S. epidermidis* INCQS 00016 (ATCC 12228) and MRSA strain INCQS 00306 (ATCC 33591). The gene target for *S. haemolyticus* was the *mvaA* gene, which plays a role in the mevalonate pathway by encoding HMG-Coa reductase, a protein responsible for the biosynthesis of isopentenyl diphosphate subunits involved in the formation of the cell wall [12]. The *coa* gene (staphylocoagulase, acetyl-CoA acethyltransferase) was targeted to detect *S. aureus*. The *mecA* gene was used for MRS strains [13,14] and ribonucleotide-diphosphate reductase (*rdr*) for *S. epidermidis* [15].

The PCR conditions for all reactions were as follows: one cycle of denaturation at 95 °C for 10 min, followed by 40 cycles of denaturation at 95 °C for 15 s and annealing at 50 °C for 1 min. The HRM step was performed immediately after PCR cycling as follows: denature at 95 °C for 10 s and anneal at 60 °C for 1 min. For melting curve step: denature at 95 °C for 15 s and anneal at 60 °C for 15 s. For HRM step: denature at 95 °C for 15 s and anneal at 60 °C for 15 s.

### 2.5. Sequencing of the mecA Gene

PCR amplification of the *mecA* gene was performed as previously described [14]. Amplification conditions were as follows: holding at 95 °C for 10 min, followed by 40 cycles of 95 °C for 15 s (denaturation), 50 °C for 1 min (annealing), 60 °C for 10 s (extension) and a final extension at 60 °C for 7 min. The products amplified were submitted to electrophoresis with a 1.5% agarose gel and analyzed by a transilluminator to detect a 500 bp amplicon (Table 1).

PCR products were purified using ExoSAP-IT (Applied Biosystems, Foster City, CA, USA) before sequencing with the BigDye Terminator v3.1 Cycle Sequencing Kit under the following conditions: initial denaturation at 96 °C for 1 min, followed by 40 cycles of denaturation at 96 °C for 10 s, annealing at 50 °C for 5 s, and extension at 60 °C for 4 min. The sequenced products were purified, precipitated, and directly analyzed using the SeqStudio Genetic Analyzer (Applied Biosystems, Foster City, CA, USA). Sequences were edited and aligned using Bioedit 7.0 and submitted to BLAST (National Center for Biotechnology Information—NCBI, Bethesda, MD, USA) for confirmation of the amplified genes.

## 3. Results

### 3.1. Distribution of Species and Resistance Profiles

Using VITEK II (BIOMERIEUX—France) and confirmatory qPCR-HRM, the isolates were identified as follows: *S. aureus* (*n* = 28; 43.8%), *S. haemolyticus* (*n* = 12; 18.8%), *S. epidermidis* (*n* = 9; 14%), *S. hominis* (*n* = 3; 4.7%), *S. capitis* (*n* = 1; 1.5%), *S. saprophyticus* (*n* = 2; 3.1%), *S. warneri* (*n* = 1; 1.5%), *S. cohnii* (*n* = 4; 6.2%), *S. lentus* (*n* = 1; 1.5%). Three coagulase-negative staphylococci (CoNS) remained unidentified (4.7%). In summary, a total of 36 (56.2%) CoNS and 17 (26.6%) MRSA were analyzed.

**Figure 1:**
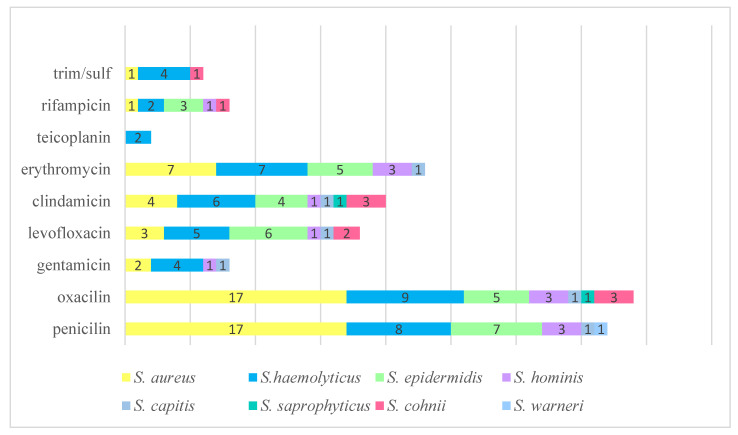
Susceptibility profiles of *Staphylococcus* spp. strains.

The antimicrobial susceptibility profiles of the *Staphylococcus* spp. isolates are detailed in Figure 1. Among these isolates, methicillin-resistant *Staphylococcus* spp. were predominant (*n* = 39; 60.9%).

VITEK II (BIOMERIEUX, Lyon, France) analysis further identified 17 multidrug-resistant strains, four of which exhibited resistance to more than three classes of antimicrobials (designated MDR5, with resistance to 5 classes, and MDR6, with resistance to 6 classes); this classification enhances the clarity of the antimicrobial resistance framework previously established. Specifically, the definition of multidrug resistance (MDR) is accompanied by the precise number of antimicrobial classes to which the bacterium exhibits resistance. Within this group, the *mecA* gene was detected in 15 isolates (Table 2).

Of the 51 *mecA*-positive strains, 28 had the gene sequenced. The analysis revealed that SCCmec type IV was predominant (*n* = 22; 73.3%), and the SCCmec type could not be determined for six strains (21%). Notably, *mecA* was present in 24 (37.5%) *S. aureus* isolates and in 10 (15.6%) *S. haemolyticus* isolates, underscoring the emerging pathogenic potential of *S. haemolyticus*.

### 3.2. High-Resolution Melting

**Figure 2:**
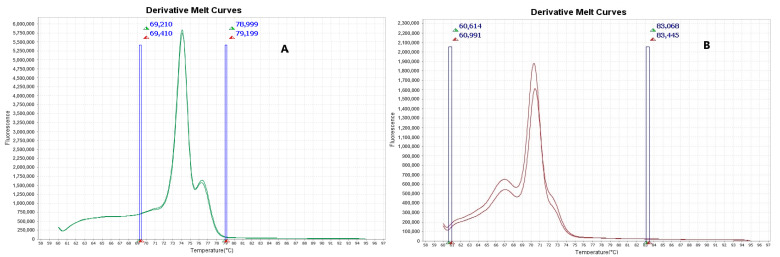

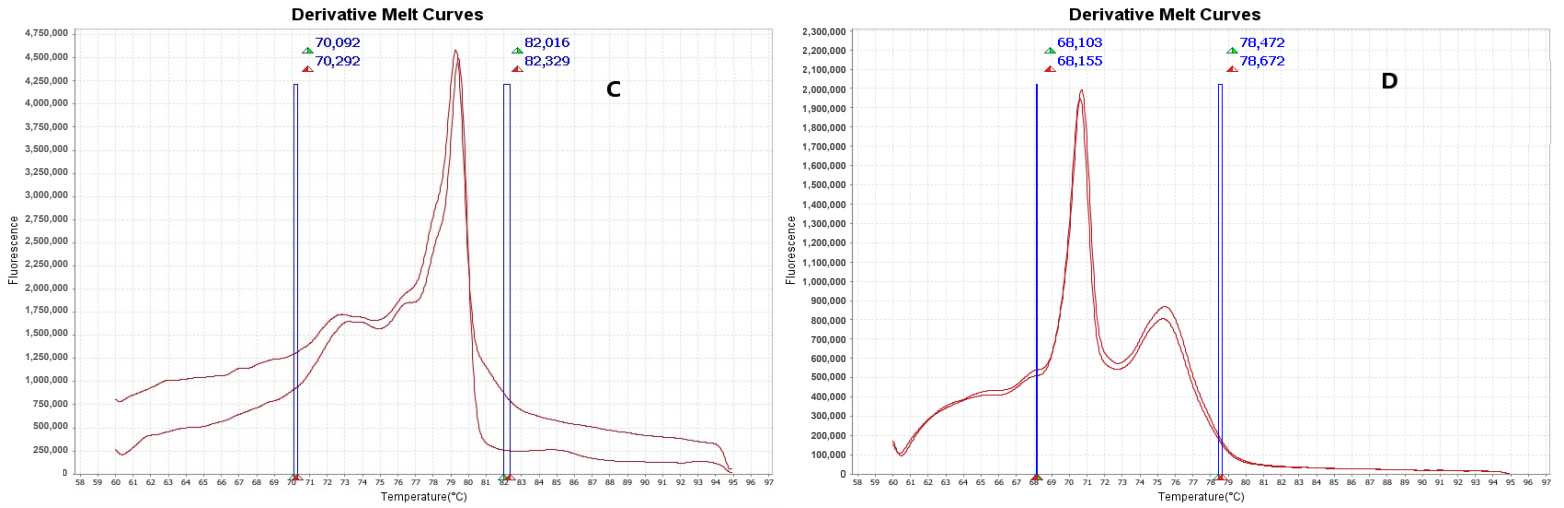
Derivative melting curves showing Tm values for (**A**) *Staphylococcus epidermidis* (74.1 °C); (**B**) *Staphylococcus aureus* (70.6 °C); (**C**) *Staphylococcus haemolyticus* (79 °C); and (**D**) *mecA* gene (70.5 °C).

*Staphylococcus* spp. strains were identified by qPCR-HRM, with specific Tm values shown in Figure 2, reported as 79 °C for *S. haemolyticus*, 70.4 °C for *S. aureus* and 74.1 °C for *S. epidermidis* using primers *Shae*, *CoaH-Ga* and *SE,* respectively. The presence of the gene *mecA* was confirmed with a Tm value of 70.5 °C using MRS primer. These Tm values were established using the following reference strains: *S. aureus* INCQS 00358 (ATCC 12600), *S. haemolyticus* CBP 98, *S. epidermidis* INCQS 00016 (ATCC 12228) and MRSA strain INCQS 00306 (ATCC 33591).

## 4. Discussion

Nosocomial infections caused by multidrug-resistant (MDR) Staphylococci have emerged as a great challenge in healthcare settings, demanding rapid and accurate identification of the etiological agents to improve patient prognosis and infection control. Traditionally, *Staphylococcus aureus* has been regarded as the primary pathogen in these infections; however, over recent decades, coagulase-negative staphylococci (CoNS) have gained prominence, particularly among immunocompromised patients. Studies such as those by Goldstein et al. (2017) [16] have underscored the increasing pathogenicity of CoNS, which now contributes significantly to the epidemiology of nosocomial infections. In our study, MRCoNS were identified in nosocomial infections, revealing an increase in antimicrobial resistance among these strains, as detected by qPCR-HRM.

Historically, *S. aureus* has been the predominant species associated with hospital-acquired infections. However, recent surveillance indicates that coagulase-negative staphylococci (CoNS), particularly *S. epidermidis* and *S. haemolyticus*, are now frequently isolated from blood cultures. Research by Lisowska-Łysiak et al. (2021) [17] underscores that both *S. aureus* and CoNS have emerged as dominant pathogens in bloodstream infections. In our study, 46.8% of the recovered strains were isolated from blood cultures, with *S. aureus* remaining the most common, followed closely by *S. epidermidis* and *S. haemolyticus*.

The rising prevalence of methicillin-resistant strains, driven by the acquisition of the *mecA* gene, further complicates the clinical scenario, as this resistance determinant is not exclusive to *S. aureus* but is also present in other *Staphylococcus* species. Notably, within four MDR *S. haemolyticus* strains identified in our study, two were classified as MDR6 carrying the *mecA* gene, in contrast to the four MDR *S. epidermidis*, of which two did not harbor the *mecA* gene, with one of these classified as an MDR5. These findings suggest that, within the CoNS group, *S. epidermidis* may not currently represent a major emerging concern regarding methicillin resistance. A study conducted across multiple European countries indicates a rising incidence of nosocomial infections caused by CoNS [17].

The rapid dissemination of multidrug-resistant strains, including those related to healthcare-associated methicillin-resistant *Staphylococcus* (HA-MRS), has significant implications for both patient outcomes and hospital infection control policies. Immunocompromised patients are particularly vulnerable, and the presence of resistant CoNS strains challenges conventional therapeutic strategies, necessitating prompt and precise diagnostic approaches.

The development of molecular techniques has revolutionized the detection and characterization of pathogens. PCR-based methods, particularly real-time PCR (qPCR-Taqman), have become the gold standard for rapid identification due to their high sensitivity, specificity, and speed. These methods enable the detection of bacterial DNA directly from clinical samples, bypassing the need for time-consuming culture techniques. While qPCR is widely used, some laboratories still rely on culture-based methods due to cost constraints. Besides speed and accuracy, qPCR-based methods can deliver results in less than two hours, which is critical for timely clinical decisionmaking, and the computer-generated outputs reduce the subjectivity often encountered in the interpretation of phenotypic tests.

However, the conventional qPCR-Taqman assay relies on the use of fluorescent probes to detect specific DNA fragments. This requirement not only increases the cost of the test but can also limit its accessibility in resource-constrained settings.

Our study highlights the implementation of qPCR coupled with High-Resolution Melting (HRM) analysis as a promising alternative to conventional qPCR-Taqman assays. The qPCR-HRM method offers several advantages since it eliminates the need for expensive fluorescent probes, making the assay more affordable, especially for developing countries. Similar to standard qPCR, HRM analysis can deliver results in a short timeframe, which is essential for early diagnosis and treatment. By analyzing the melting profiles of the amplified DNA fragments, qPCR-HRM can distinguish between closely related species such as *S. aureus*, *S. epidermidis*, and *S. haemolyticus*, thereby minimizing misidentification. Also, this approach allows for the rapid screening of antimicrobial resistance (AMR) genes, such as *mecA*, which is crucial for the early detection of MDR strains. However, while qPCR-HRM is a powerful and rapid technique for detecting genetic variations, including antimicrobial resistance genes, it has some limitations. (a) It requires high-quality, purified DNA; (b) low DNA concentrations may lead to poor amplification and unreliable melting curve analysis.

The integration of qPCR-HRM into routine diagnostics can significantly enhance the ability to monitor nosocomial infections, enabling healthcare providers to implement targeted interventions more effectively.

Understanding the molecular epidemiology of methicillin resistance in *Staphylococcus* spp. is vital for infection control. SCCmec typing differentiates between various methicillin-resistant clones. SCCmec Types I, II, and III are traditionally allied with healthcare-associated MRSA (HA-MRSA), reflecting a background of multidrug resistance. SCCmec Types IV and V are more commonly found in community-associated MRSA (CA-MRSA); however, recent evidence suggests that these types are increasingly present in nosocomial settings. Studies in Latin America have reported a high prevalence of SCCmec type IV among *mecA*-positive strains, indicating its diffusion in both community and hospital environments [18]. Our findings corroborate this trend, underscoring the necessity of incorporating SCCmec typing into epidemiological surveillance protocols to better track and manage the spread of resistant strains.

According to a study by Moreno et al. (2020) [19], resources for epidemiological surveillance of MRSA in Latin America are limited, resulting in significant data gaps, particularly in regions lacking extensive microbiological surveillance facilities. Despite these limitations, MRSA remains a leading cause of nosocomial infections, with prevalence rates ranging from 6% in Cuba to 70% in Costa Rica and 80% in Chile and Peru [20].

The rapid spread of MDR *Staphylococcus*, including both *S. aureus* and CoNS, poses a significant threat to patient safety and public health. Implementing advanced molecular diagnostics like qPCR-HRM can play a critical role in early detection and intervention, facilitating prompt therapeutic decisions to improve patient outcomes, enabling continuous monitoring of resistance patterns and the distribution of SCCmec types and offering a financially sustainable diagnostic tool that can be widely adopted, particularly in resource-limited settings.

Looking ahead, future research should focus on further refining qPCR-HRM techniques, integrating them with comprehensive genomic surveillance systems, and exploring additional molecular markers that could enhance diagnostic precision. Moreover, the development of point-of-care devices based on HRM analysis could further revolutionize the rapid detection and management of nosocomial infections. Currently, qPCR-HRM is widely used in various studies for diagnostic applications and strain genotyping, enabling the differentiation of multiple microorganisms [21,22,23].

In summary, the increasing incidence of nosocomial infections caused by MDR Staphylococci necessitates a paradigm shift in diagnostic methodologies. The use of qPCR-HRM presents a robust, cost-effective, and rapid approach that not only facilitates the accurate identification of pathogens but also supports effective infection control measures, thereby contributing to improved patient prognoses and the overall management of healthcare-associated infections.

## 5. Conclusions

Antimicrobial resistance (AMR) is an evolutionary process among microorganisms. The indiscriminate use of antimicrobials contributes to the development of resistance among species, becoming a threat to public health [24].

Subsequent to the use of methicillin in clinical practice in 1960, MRSA was first identified. Over the years, methicillin resistance has risen in hospital and community settings, becoming a burden on the treatment of infections [25]. In addition, the pathogen–drug combination that leads to an increase in deaths attributable to AMR globally is *Staphylococcus aureus* associated with methicillin resistance [26]. In our study, among 64 Staphylococci strains, only 11 were susceptible to methicillin, showing the continuous emergence of resistant strains. Despite the small sample size, we identified a significant percentage of MRS strains among the isolates, particularly MRCoNS. Additionally, qPCR-HRM proved to be a promising and cost-effective alternative for low-resource hospitals, offering a viable substitute for more expensive methods such as qPCR-TaqMan, MALDI-TOF, and DNA sequencing-based techniques.

## Figures and Tables

**Table 1:** Genes, primer sequences, product sizes (bp) and respective references.

Gene	Primer	Sequence	Size (bp)	Reference
*coa*	*CoaH-Ga*	F: 5′-AGTCTTAAACATAACATGACCTAA-3′ R: 5′-TTATTGCTGTGATTGTCGTAT-3′	89	This study
*rdr*	*SE*	F: 5′-ATCAAAAAGTTGGCGAACCTTTTCA-3′	124	Martineau et al., 1996 [15]
		R: 5′-CAA AAGAGCGTGGAGAAAAGTATCA-3′		
*mvaA*	*Shae*	F: 5′- GGTCGCTTAGTCGGAACA AT-3′	271	Schuenck et al., 2007 [12]
		R: 5′-CACGAGCAATCTCATCACCT-3′		
*mecA*	*MRS*	F: 5′-TAGAAATGACTGAACGTCCG-3′	154	Santos et al., 1999 [13]
		R: 5′-TTGCGATCAATGTTACCGTAG-3′		
*mecA*	*mecA*	F: 5′-GATCTGTACTGGGTTAATCA-3′	500	Breves et al., 2005 [14]
		R: 5′-CATATGACGTCTATCCATTT3-3′		

bp: base pairs, *coa*: staphylocoagulase enzyme gene, *rdr*: ribonucleotide-diphosphate reductase gene, *mvaA*: HMG-Coa reductase enzyme gene, *mecA*: penicillin binding protein 2 gene.

**Table 2:** Multidrug-resistant (MDR) strains carrying *mecA* gene.

Strain	Species	MDR	*mecA*
43	*Staphylococcus haemolyticus*	+	+
72	*Staphylococcus epidermidis*	+	+
98	*Staphylococcus haemolyticus*	MDR6	+
111	*Staphylococcus epidermidis*	+	+
115	*Staphylococcus haemolyticus*	MDR6	+
118	*Staphylococcus epidermidis*	+	-
145	*Staphylococcus aureus*	+	+
146	*Staphylococcus haemolyticus*	+	+
155	*Staphylococcus aureus*	+	+
159	*Staphylococcus cohnii*	+	+
160	*Staphylococcus epidermidis*	MDR5	-
162	*Staphylococcus aureus*	+	+
166	*Staphylococcus aureus*	+	+
176	*Staphylococcus aureus*	+	+
179	*Staphylococcus hominis*	+	+
211	*Staphylococcus saprophyticus*	+	+
225(2)	*Staphylococcus aureus*	MDR5	+

Results obtained by VITEK II (BIOMERIEUX—France) in comparison with Polymerase Chain Reaction (PCR). MDR5: resistant to 5 classes of antimicrobials, MDR6: resistant to 6 classes of antimicrobials.

## Data Availability

Data are contained within the article.
